# Prevention and Management of Pediatric Sensitive Skin Conditions in India: A Modified Delphi Consensus

**DOI:** 10.7759/cureus.116261

**Published:** 2026-09-15

**Authors:** Neeraj Gupta, Medhu Rengasamy, R Kishore Kumar, Giridhar Sethuraman, K. P Kumar, Mukesh Sanklecha, Rahul Verma, Manish Mittal, Vinay Joshi, Suresh Natarajan, Joshi A Kerketta, Tanvi Pal, Resham Vasani, Ramkumar Ramamoorthy, Sneha Menon, Priti Thakor, Isha Naik, Vijay Chamle, Nikhil Bangale, Aanchal Gvalani

**Affiliations:** 1 Department of Pediatrics, Sir Ganga Ram Hospital, Delhi, IND; 2 Department of Dermatology, Madras Medical College, Chennai, IND; 3 Department of Pediatrics, Cloudnine Group of Hospitals, Bangalore, IND; 4 Department of Pediatrics, Chettinad Hospital and Research Institute and Neolife Children's Hospital, Chennai, IND; 5 Department of Pediatrics, MNR Medical College, Hyderabad, IND; 6 Department of Pediatrics, Bombay Hospital, Mumbai, IND; 7 Department of Pediatrics, Sir HN Reliance Foundation Hospital, Mumbai, IND; 8 Department of Pediatrics, Cocoon Hospital, Jaipur, IND; 9 Department of Pediatrics, Kokilaben Dhirubhai Ambani Hospital, Mumbai, IND; 10 Department of Pediatrics, Southern Railway Hospital, Perambur, Chennai, IND; 11 Department of Pediatrics, The Mission Hospital, Durgapur, IND; 12 Department of Dermatology, BLK Max Hospital and Kubba Clinic, Delhi, IND; 13 Department of Dermatology, Bhojani Clinic and BJ Wadia Hospital for Children, Mumbai, IND; 14 Department of Dermatology, Kanchi Kamakoti Childs Trust Hospital, Chennai, IND; 15 Department of Medical Safety Sciences, JNTL Consumer Health (India) Pvt. Ltd., Mumbai, IND; 16 Department of Medical Safety Sciences, JNTL Consumer Health (India) Pvt. Ltd., Pune, IND

**Keywords:** colloidal oatmeal, diagnosis, emollients, management, pediatric, sensitive skin

## Abstract

Sensitive skin poses significant dermatological challenges in the pediatric population, necessitating thorough exploration and understanding. In this study, we aim to provide neonatologists, pediatricians, dermatologists, and allergists in India with consensus-based recommendations for the prevention and management of sensitive skin in the pediatric age group. A modified Delphi method was used to arrive at a consensus. A group of 15 experts with experience in pediatric skincare constituted the panel. Panel members representing diverse specialties from the major regions of India participated in the consensus process. Statements were developed based on a structured review of the literature identified through PubMed/Medical Literature Analysis and Retrieval System Online (MEDLINE) (January 2000 to May 2024; updated in March 2025) using the keywords "sensitive skin," "pediatric," "emollient," "prevention," and other related terms*.* Ninety-six clinically relevant questions and/or statements were drafted for discussion: (i) burden, pathophysiology, and challenges in the diagnosis of sensitive skin; (ii) symptoms, risk factors, and consequences; (iii) clinical diagnosis; (iv) prevention and management; and (v) the positioning of colloidal oatmeal-based emollients in managing and preventing sensitive skin in the pediatric population.

The consensus level was classified into agreement (mean score of ≥4), no consensus, and exclusion (mean score of <4 after the second round). The process involved two rounds of Delphi surveys and one round of discussion. The experts unanimously agreed that diaper dermatitis, xerosis, and atopic dermatitis (AD) were the most prevalent sensitive skin conditions in children. Physical examinations and assessments of clinical and medical histories were the most effective primary diagnostic methods for identifying sensitive skin. The experts recommended moisturizers/emollients as a cornerstone for managing sensitive skin conditions and enhancing skin health and quality of life (QoL). Emollients should hydrate the skin, repair the skin barrier, and restore ceramide and microbiome balance, all while avoiding irritants. The experts recommended colloidal oatmeal-based emollients in xerosis and AD and as supportive adjunctive care alongside allergen avoidance in allergic contact dermatitis because of their prebiotic properties, soothing effects, and ability to activate endogenous ceramide production, which helps restore the skin's barrier and integrity. In the panel's view, the early and long-term usage of colloidal oatmeal-based emollients helps in the reduction of flare severity and frequency and, potentially, reliance on corticosteroids.

## Introduction

Sensitive skin is a significant clinical concern characterized by heightened reactivity to environmental, chemical, and physical factors [1,2]. A key feature of sensitive skin is the disruption of the skin barrier, leading to increased transepidermal water loss (TEWL) and xerosis [3]. As defined by the special interest group on sensitive skin of the International Forum for the Study of Itch (IFSI), sensitive skin is a multifaceted syndrome where individuals experience unpleasant sensations, such as stinging, burning, and itching, in response to stimuli that normally should not provoke such sensations [4-6].

Sensitive skin is associated with a wide spectrum of conditions, such as atopic dermatitis (AD), contact dermatitis, psoriasis, and rosacea [7,8]. AD often manifests as a chronic, relapsing inflammatory condition, typically accompanied by intense pruritus. Furthermore, 90% of cases emerge within the first five years of life [6]. In pediatric patients, the impact of sensitive skin extends beyond mere physical discomfort. It poses significant implications for children's overall well-being, affecting their physical and emotional health [9,10]. This condition, often marked by symptoms such as erythema, dryness, pruritus, and discomfort, can profoundly affect the quality of life (QoL) of children and their caregivers [5,10,11]. Sleep disturbances affect up to 60% of children with AD, with this figure rising to 83% during flare-ups [12]. Older children with AD may also face bullying and social avoidance, which can lead to diminished self-esteem, social withdrawal, and feelings of rejection [11].

In India, distinct environmental and cultural factors contribute to the unique presentation and management challenges of sensitive skin in the pediatric population [1,8,13]. The pathophysiology of sensitive skin is complex and multifactorial, involving an impaired skin barrier function, altered immune responses, and heightened sensory perception [1]. These factors can be exacerbated by environmental triggers, such as extreme temperatures, pollution, and the use of harsh personal care products, all of which are common in India. The primary objectives of sensitive skin management should be to enhance the patients' QoL while minimizing the potential adverse effects associated with therapy [1,13]. Targeting these two key areas is critical for achieving optimal outcomes, particularly in patients with moderate-to-severe forms of the condition.

The effective management of sensitive skin involves gentle skincare, including the use of mild cleansers and well-tolerated emollients, while minimizing triggers and addressing psychosocial factors [14-17]. Furthermore, educating patients about the underlying causes and implementing a comprehensive skincare plan are essential components of successful management. Existing recommendations on sensitive skin are largely based on adult populations or from international expert panels whose environmental, cultural, and product-access contexts differ substantially from those encountered in Indian practice. The recent Indian position statement on sensitive skin [1] addresses the condition across the age spectrum rather than the pediatric population specifically, while the Indian guidelines on AD [18] address a specific diagnosis rather than the broader sensitive skin spectrum. Furthermore, they do not provide pediatric-specific, practice-level guidance on prevention and daily skincare for Indian children. The present consensus is intended to complement the existing guidelines by focusing specifically on the pediatric age group and on practical, India-relevant prevention and management.

For the purpose of this document, sensitive skin is understood as defined above (by IFSI) and is conceptually distinct from established diagnoses such as AD, allergic contact dermatitis, diaper dermatitis, xerosis, and psoriasis, each of which has its own diagnostic framework. In routine Indian pediatric practice, sensitive skin frequently overlaps with these conditions. Accordingly, this consensus does not treat them as a single diagnostic category; instead, it focuses on the shared principles of skin barrier protection and gentle skincare applicable across this clinical spectrum in children. This consensus therefore aims to guide clinicians in everyday practice rather than to serve as a product-specific recommendation.

## Materials and methods

Panel composition and selection

A panel of 15 experts with significant experience in pediatric skincare participated in developing this expert consensus (Figure 1). The panel members were selected purposively from across the major regions of India on the basis of documented clinical and academic expertise in pediatric dermatology and pediatrics; the panel comprised both pediatricians and dermatologists to ensure multidisciplinary and geographic representation, and their affiliations are listed in the author information. The composition of the panel is available as a supplementary file (see Appendices).

**Figure 1:**
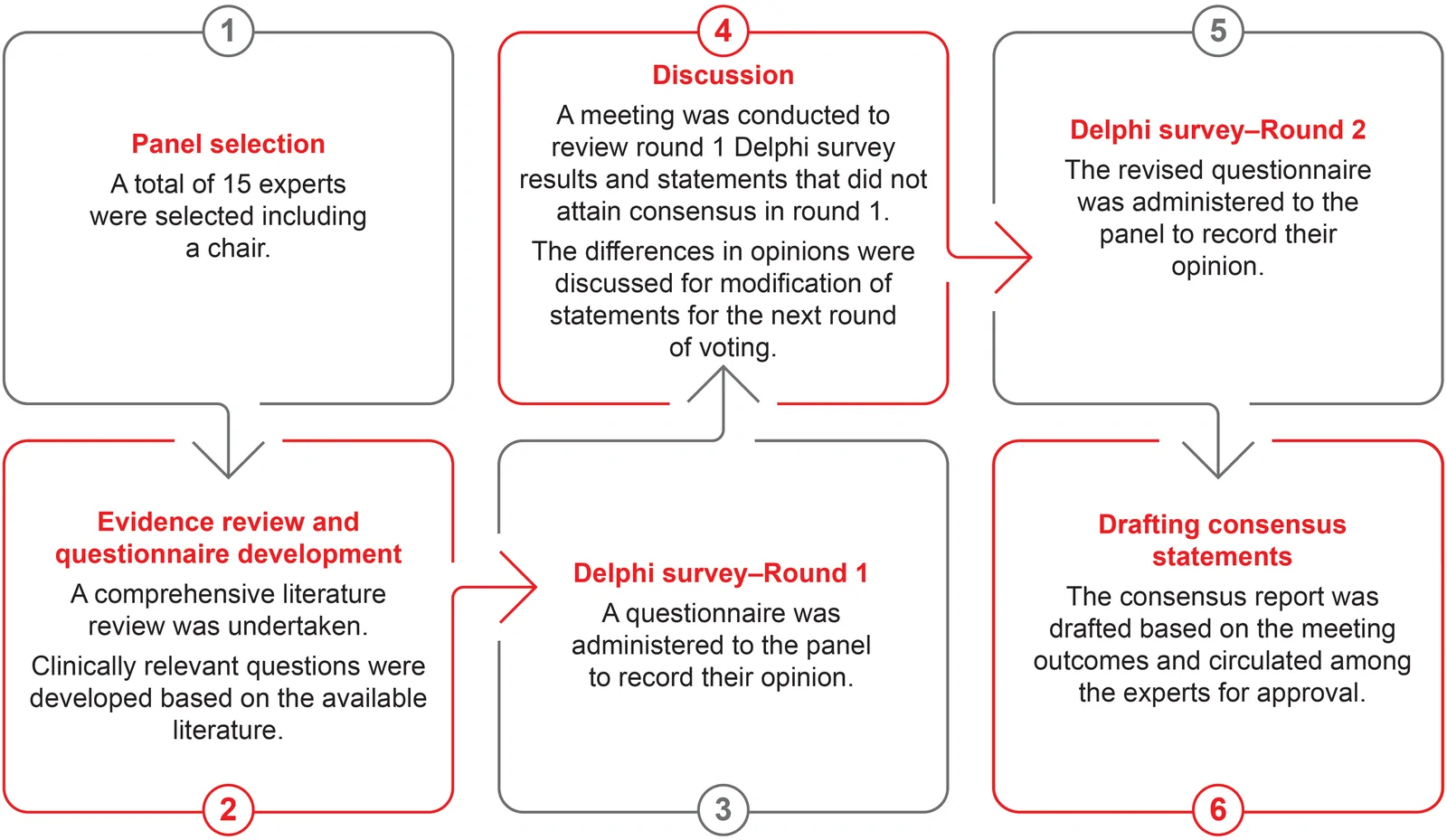
Study methodology Source: image created by the authors using Adobe Illustrator (version 30.3; Adobe Inc., San Jose, CA)

Evidence review

An electronic search of the PubMed/Medical Literature Analysis and Retrieval System Online (MEDLINE) database was conducted covering January 2000 to May 2024, with an updated search performed in March 2025. Search terms included "sensitive skin," "pediatric," "children," "diagnosis," "management," "emollient," and "prevention," combined using the Boolean operators "AND" and "OR" with appropriate variations in search phrasing. Randomized controlled trials, case reports, practice guidelines, consensus recommendations, surveys, systematic literature reviews, and meta-analyses were eligible for inclusion; studies involving animals and publications in languages other than English were excluded, and duplicate records were removed. The search was undertaken as a pragmatic narrative evidence review to inform the development of discussion items rather than as a systematic review. Accordingly, staged record counts and a PRISMA flow diagram were not generated, and the retrieved literature was used to frame statements for panel consideration rather than to derive graded recommendations.

Questionnaire development

A total of 96 clinically relevant questions and statements were drafted by the steering group (identified by the panel) on the basis of the literature review and circulated to the full panel for their voting (see Appendices). The questionnaire was not subjected to formal external pilot testing before administration. Supporting evidence was drawn from the literature review as a whole to frame the items rather than assigned to individual statements; the relevant literature is cited throughout the discussion. Of these, 52 were statements submitted for formal consensus voting, whereas 44 were descriptive opinion-survey items used to characterize clinical practice patterns and were therefore not subjected to consensus voting. The questionnaire was organized into the following domains: (1) burden, pathophysiology, and key challenges in the accurate diagnosis of sensitive skin; (2) clinical signs, symptoms, risk factors, and consequences; (3) the diagnosis of sensitive skin and conditions in the pediatric population; (4) the prevention and management of sensitive skin in the pediatric population; and (5) the role and positioning of colloidal oatmeal-based emollients in managing and preventing sensitive skin in the pediatric population. These five domains were defined a priori by the panel before item development and before voting. The number of statements arising within each domain reflects the number of distinct questions considered by the panel and not a weighting applied to the consensus as a whole.

Delphi process and consensus criteria

A modified Delphi method was used to arrive at a consensus [19]. Voting was conducted electronically and independently, and individual responses were anonymized to encourage candid feedback. All 15 experts participated in round 1; 14 of 15 participated in round 2. An electronic survey link to these questions was sent to all the participants to record their views (Delphi survey, round 1). The level of consensus was categorized as agreement (mean or average score of ≥4 on the five-point Likert scale), no consensus (mean or average score of <4 on the five-point Likert scale), and exclusion (mean or average score of <4 on the five-point Likert scale after two rounds) (Table 1) [20-22]. As a post hoc sensitivity analysis, the consensus set was additionally tested against a percentage-agreement threshold of ≥80% of participating experts rating a statement 4 or 5. Mean scores are reported with the standard deviation of the panel ratings for each statement (Table 2).

**Table 1:** Criteria for consensus Consensus criteria modified and adapted from Rüetschi and Salazar (2022) [20], Rüetschi and Salazar (2020) [21], and Burgess Kelleher et al. (2020) [22]

Consensus criteria	
Consensus/agreement	The mean/average score is ≥4 on the five-point Likert scale
No consensus	The mean/average score is <4 on the five-point Likert scale
Exclusion	After two rounds, the statement is excluded if the mean/average score is <4 on the five-point Likert scale

**Table 2:** Summary of statements that achieved consensus (N = 45) Source: original table developed by the authors *One expert did not participate in the second round of voting **Stimuli refer to multiple factors, which may be physical, chemical, allergenic, and occasionally psychological (stress) or hormonal AD, atopic dermatitis; FLG, filaggrin; NF-kB, nuclear factor kappa B; SD, standard deviation

Consensus statements		Number (%) of experts strongly agreeing or agreeing with the statement/mean score (round 1)			Number (%) of experts strongly agreeing or agreeing with the statement/mean score (round 2)*		
		Strongly agree, n (%)	Agree, n (%)	Mean score (SD)	Strongly agree, n (%)	Agree, n (%)	Mean score (SD)
Definition and impact							
1	Sensitive skin is defined as a condition where the skin is more prone to the occurrence of unpleasant sensations (dryness, pruritus, burning, stinging, pain, warmth, and/or erythema) in response to stimuli** that otherwise should not provoke such sensations, which could be self-reported by older children or based on parental perceptions for younger children.	No consensus	No consensus	No consensus	7 (50%)	7 (50%)	4.5 (0.52)
2	Sensitive skin conditions, including AD, in pediatric patients often have a substantial impact on their quality of life, such as their mood and ability to go back to sleep.	10 (66.67%)	5 (33.33%)	4.67 (0.49)	Not applicable	Not applicable	Not applicable
3	Sensitive skin may originate from an altered stratum corneum, resulting in an imbalance in the regulation of water homeostasis.	7 (46.67%)	8 (53.33%)	4.47 (0.52)	Not applicable	Not applicable	Not applicable
4	Considering the studies that have shown only a fraction (few to 50%) of patients carry FLG mutations and 60% of these carriers do not develop AD, FLG mutation is not the only major cause of sensitive skin and conditions such as AD.	6 (40%)	9 (60%)	4.40 (0.51)	Not applicable	Not applicable	Not applicable
5	Sensitive skin syndrome is associated with low skin hydration in infants and children.	9 (60%)	4 (26.67%)	4.33 (1.11)	Not applicable	Not applicable	Not applicable
6	The level of awareness among parents and caregivers regarding sensitive skin in the pediatric age group is progressively increasing/better compared with that in the yesteryears.	No consensus	No consensus	No consensus	7 (50%)	7 (50%)	4.5 (0.52)
Presentation of symptoms, clinical signs, risk factors, and consequences							
7	The pediatric population with sensitive skin exhibits more clinical signs of skin dryness compared with the population without sensitive skin.	8 (53.33%)	7 (46.67%)	4.53 (0.52)	Not applicable	Not applicable	Not applicable
8	Sensitive skin can serve as a cutaneous manifestation of underlying skin disorders.	7 (46.67%)	8 (53.33%)	4.47 (0.52)	Not applicable	Not applicable	Not applicable
9	A history of childhood AD is more frequent in children with sensitive skin, and children with sensitive skin often report a concomitant atopic condition.	8 (53.33%)	6 (40%)	4.47 (0.64)	Not applicable	Not applicable	Not applicable
10	FLG mutation and reduction in FLG levels are not the only causes of AD, and additional factors in the development of AD include environmental factors, other genetic predispositions, biochemical changes, and immune dysfunction.	9 (60%)	6 (40%)	4.60 (0.51)	Not applicable	Not applicable	Not applicable
11	Allergen sensitization through a defective skin barrier can lead to clinical diseases or predispose individuals to the development of AD and food allergies.	7 (46.67%)	7 (46.67%)	4.40 (0.63)	Not applicable	Not applicable	Not applicable
Diagnosis of sensitive skin conditions, including AD, in the pediatric population							
12	In pediatric patients, sensitive skin conditions, including AD, should be diagnosed based on a comprehensive evaluation, considering both subjective symptoms reported by the child or parent/caregiver and objective clinical signs observed by healthcare providers.	8 (53.33%)	6 (40%)	4.47 (0.64)	Not applicable	Not applicable	Not applicable
13	Diagnosis of sensitive skin is primarily through the observation of objective clinical signs, which include redness, dryness, scaling, and peeling, along with sensations of itching, burning, tightness, stinging, and pain.	8 (53.33%)	7 (46.67%)	4.53 (0.52)	Not applicable	Not applicable	Not applicable
Management and prevention of sensitive skin conditions in the pediatric population							
14	Moisturizers/emollients play a crucial role in the daily management of individuals with sensitive skin.	11 (73.33%)	4 (26.67%)	4.73 (0.46)	Not applicable	Not applicable	Not applicable
15	The right emollient can not only restore but also repair the skin barrier by stimulating endogenous ceramide production.	5 (33.33%)	7 (46.67%)	4.13 (0.74)	Not applicable	Not applicable	Not applicable
16	While the exogenous fortification of ceramides only helps replenish depleted ceramides, the activation of endogenous ceramide production helps restore the natural ceramide balance of the skin.	5 (33.33%)	8 (53.33%)	4.20 (0.68)	Not applicable	Not applicable	Not applicable
17	Emollients used in the management of sensitive skin in the pediatric population should be free from potentially irritating substances and should undergo thorough fragrance evaluation for allergenicity and irritant potential.	9 (60%)	6 (40%)	4.60 (0.51)	Not applicable	Not applicable	Not applicable
18	Considering that all emollients are not equal, different types of emollients exhibit diverse physiological effects on the skin barrier; in subjects with sensitive skin conditions, including AD, a written recommendation of emollients should be preferred over an oral recommendation.	9 (60%)	6 (40%)	4.60 (0.51)	Not applicable	Not applicable	Not applicable
19	Skin rehydration and skin barrier repair with regular and long-term usage of emollients reduce the need for other topical treatments.	8 (53.33%)	7 (46.67%)	4.53 (0.52)	Not applicable	Not applicable	Not applicable
20	The early and regular use of emollients that stimulate/activate endogenous production of ceramides to help restore the skin's natural ceramide balance in infants and children with sensitive skin conditions, including AD, extends the duration between flares and reduces the intensity and frequency of flares, irrespective of the severity of the condition.	6 (40%)	6 (40%)	4.20 (0.77)	Not applicable	Not applicable	Not applicable
21	Emollients should be applied to damp skin soon after bathing for maximum benefits (i.e., better penetration).	10 (66.67%)	5 (33.33%)	4.67 (0.49)	Not applicable	Not applicable	Not applicable
22	The management of AD should not adopt a "one-size-fits-all" approach, and treatment should be tailored to individual reactivity patterns.	11 (73.33%)	3 (20%)	4.60 (0.83)	Not applicable	Not applicable	Not applicable
23	For pediatric patients with AD, it is advisable to bathe in lukewarm water for 5-10 minutes, use a moisturizing cleanser, gently pat the skin dry, and then apply emollients liberally.	11 (73.33%)	4 (26.67%)	4.73 (0.46)	Not applicable	Not applicable	Not applicable
24	The early initiation of emollient usage is recommended from day 1 (day 1 of diagnosis or day 1 of appearance of symptoms or increase in severity or day 1 of life in all high-risk infants or in all babies) for the management of sensitive skin conditions as it is considered the cornerstone of the management paradigm; hence, in clinical practice, regular emollient usage for improving signs and symptoms of sensitive skin, including AD, and tolerability in children and infants is recommended.	8 (53.33%)	5 (33.33%)	4.33 (0.90)	Not applicable	Not applicable	Not applicable
25	Recommendations for applying emollients for managing AD in children typically involve twice-daily use, but this frequency can differ based on individual and environmental factors, as well as the degree of skin dryness.	9 (60%)	6 (40%)	4.60 (0.51)	Not applicable	Not applicable	Not applicable
26	The use of an emollient in addition to skin hydration plays a role in restoring the skin barrier integrity by upregulating the enzymes and genes downregulated in AD/sensitive skin.	7 (46.67%)	8 (53.33%)	4.47 (0.52)	Not applicable	Not applicable	Not applicable
Role and positioning of colloidal oatmeal-based emollients in managing and preventing sensitive skin in the pediatric population							
27	The regular use of colloidal oatmeal-based emollients has the potential to prevent allergen penetration/sensitization and subsequently prevent the progression of the condition (which may include the progression of atopic march).	7 (46.67%)	7 (46.67%)	4.33 (0.82)	Not applicable	Not applicable	Not applicable
28	Colloidal oatmeal-based moisturizers should be recommended for pediatric patients with sensitive skin due to their multitherapeutic benefits, such as prebiotic properties, overall soothing effects, and the ability to stimulate/activate endogenous ceramide production, which help in restoring skin integrity.	7 (46.67%)	7 (46.67%)	4.40 (0.63)	Not applicable	Not applicable	Not applicable
29	Colloidal oatmeal-based emollients can be used as early as the first week of life to prevent AD, maintain skin barrier integrity, and reduce the risk of sensitive skin conditions, especially in high-risk infants.	6 (40%)	4 (26.67%)	4.00 (1.00)	Not applicable	Not applicable	Not applicable
30	Regular prophylactic application of colloidal oatmeal-based emollients, initiated in early infancy in high-risk infants, helps prevent the occurrence and/or reduce the severity of AD.	No consensus	No consensus	No consensus	3 (21.43%)	9 (64.29%)	4.07 (0.62)
31	Colloidal oatmeal-based emollients can nourish and protect a baby's delicate and fragile skin and limit the progression of AD.	6 (40%)	5 (33.33%)	4.13 (0.83)	Not applicable	Not applicable	Not applicable
32	Colloidal oatmeal restores and repairs the skin barrier by not only forming a protective hydrolipid film on the skin but also enhancing the water-binding and moisture-retention properties of the stratum corneum.	6 (40%)	8 (53.33%)	4.33 (0.62)	Not applicable	Not applicable	Not applicable
33	Colloidal oatmeal-based emollients are clinically effective in managing sensitive skin, including atopic skin conditions, in children and are well-tolerated.	7 (46.67%)	6 (40%)	4.27 (0.88)	Not applicable	Not applicable	Not applicable
34	Colloidal oatmeal-based emollients should be recommended as part of core management from day 1 for sensitive skin conditions, including atopic skin, to moisturize the skin, repair the skin barrier, and help relieve symptoms.	6 (40%)	7 (46.67%)	4.20 (0.86)	Not applicable	Not applicable	Not applicable
35	The initial treatment strategy for sensitive skin conditions, including AD in children, should involve applying a colloidal oatmeal-based barrier repair emollient with or without standard-of-care treatment to restore and repair the skin barrier while maintaining natural skin homeostasis.	7 (46.67%)	6 (40%)	4.20 (1.08)	Not applicable	Not applicable	Not applicable
36	Skin rehydration and repair with colloidal oatmeal-based emollients potentially reduce the requirement for topical corticosteroids and other anti-inflammatory agents.	8 (53.33%)	4 (26.67%)	4.33 (0.82)	Not applicable	Not applicable	Not applicable
37	The routine application of colloidal oatmeal-based emollients on infant skin can alleviate signs of irritation and discomfort associated with dryness.	6 (40%)	8 (53.33%)	4.33 (0.62)	Not applicable	Not applicable	Not applicable
38	Applying a colloidal oatmeal-based emollient twice daily combined with a colloidal oatmeal-based cleanser to bathe babies and young children with AD can significantly improve symptoms such as itching, dryness, roughness, and overall disease severity.	7 (46.67%)	5 (33.33%)	4.13 (1.13)	Not applicable	Not applicable	Not applicable
39	Colloidal oatmeal-based emollients have multitherapeutic beneficial effects on the skin barrier through the enhancement of barrier homeostasis and are thus a total package for the management of sensitive skin conditions, including AD.	7 (46.67%)	6 (40%)	4.27 (0.88)	Not applicable	Not applicable	Not applicable
40	Colloidal oatmeal-based emollient therapy helps reduce the clinical manifestations of AD, including dryness, pruritus, erythema, scaling, and roughness in neonates, infants, and children.	5 (33.33%)	9 (60%)	4.27 (0.59)	Not applicable	Not applicable	Not applicable
41	Colloidal oatmeal-based moisturizers should be the cornerstone for the effective management of sensitive skin conditions, including AD, and can be recommended for children with xerosis and AD.	5 (33.33%)	8 (53.33%)	4.20 (0.68)	Not applicable	Not applicable	Not applicable
42	Colloidal oatmeal-based moisturizers can be the cornerstone for the effective management of sensitive skin conditions, including AD, and can be recommended for children with allergic contact dermatitis.	5 (33.33%)	7 (46.67%)	4.13 (0.74)	Not applicable	Not applicable	Not applicable
43	Colloidal oatmeal-based emollients can significantly reduce the intensity of pruritus in children with sensitive skin conditions, including AD.	5 (33.33%)	8 (53.33%)	4.20 (0.68)	Not applicable	Not applicable	Not applicable
44	Colloidal oatmeal-based emollients can help break the itch-scratch cycle due to the presence of avenanthramides, which inhibit neurogenic inflammation, the NF-kB pathway, and histamine signaling.	6 (40%)	8 (53.33%)	4.33 (0.62)	Not applicable	Not applicable	Not applicable
45	Colloidal oatmeal-based emollients can stimulate the production of not only FLG but also other key skin barrier biomarkers and genes involved in keratinocyte differentiation, lipid production, and tight junction formation.	5 (33.33%)	7 (46.67%)	4.13 (0.74)	Not applicable	Not applicable	Not applicable

A virtual meeting was conducted to review the round 1 Delphi survey results and statements that did not attain consensus. During this discussion, the round 1 results were reviewed, and non-consensus statements were reworded to improve clarity before being put to a second round of anonymized voting. The original wording of all items and the reworded versions of the statements taken forward to round 2 are provided in Table 2. Statements that achieved consensus in round 1 were not revised. All statements are reported exactly as voted by the panel and were not subject to post hoc editorial modification. A round 2 survey included statements that did not achieve consensus in the first round. The consensus report was drafted based on the meeting outcomes, and the final draft was emailed to all experts for approval.

## Results

Following the results of the evidence search, 52 statements and 44 descriptive survey-type items were drafted and included in the Delphi round 1 survey. The experts based their decisions on the available evidence and clinical expertise. In round 1, consensus was reached for 42 of 52 statements. The remaining 10 statements that did not achieve consensus after the first round were discussed during the meeting and modified for the second round of voting. Seven statements did not achieve consensus after round 2 of voting and were excluded (see Appendices). The finalized consensus statements (N = 45) are listed in Table 2. Percentages attributed to the descriptive survey items in the sections below describe the distribution of expert opinion and should not be interpreted as consensus outcomes. Further, in a post hoc sensitivity analysis, 43 of the 45 statements also met a ≥80% agreement threshold. Only statements 29 and 31 fell below this threshold, with agreement levels of 66.7% and 73.3%, respectively.

## Discussion

Across the five domains, the highest and most consistent levels of agreement were observed for the fundamentals of pediatric skincare. The panel reached near-unanimous agreement that sensitive skin and related barrier-compromise conditions should be diagnosed through comprehensive clinical assessment integrating reported symptoms with observed signs. The panel also agreed that moisturizers and emollients are central to daily management and should be free of potentially irritating substances and appropriately evaluated for fragrance and further agreed on the role of colloidal oatmeal-based emollients within this framework. The panel highlighted that bathing should be brief and in lukewarm water, emollients should be applied to damp skin, and caregiver education in this regard and treatment adherence are essential components of effective care. Collectively, these principles define the practical clinical framework emerging from this consensus.

Understanding the burden of sensitive skin conditions in the pediatric population

Sensitive skin is a common condition in India, affecting approximately 32% of the population [8]. Dermatological problems account for ≥30% of all outpatient visits to pediatricians, and approximately 30% of all visits to dermatologists involve pediatric patients [23]. Sensitive skin can present with a variety of distressing symptoms and significantly impact a patient's QoL. The skin of infants and children is softer and smoother than that of adults, making it more susceptible to sensitivity and inflammation [24]. Sensitive skin can affect all parts of the body, with a particular tendency to be noticeable on the face, especially around the nasolabial folds [7]. Erythema and pruritus are common concerns associated with sensitive skin, often leading to chronic scratching, which can exacerbate skin damage and impede recovery [1]. Dryness and scaling are also predominant, reflecting a compromised skin barrier with reduced moisture retention [1]. Patients frequently report burning or stinging sensations, particularly when exposed to certain topical agents or environmental factors [1]. Inflammation can also lead to localized swelling, further intensifying discomfort [1].

Sensitive skin is triggered by multiple factors that can heighten skin reactions. Environmental factors (cold, heat, sun, pollution, and moisture) can trigger sensitive skin conditions [25]. Additionally, topical applications of a wide range of cosmetic products, hair care products, and hair dyes containing allergens can result in transient skin reactions [1,26]. Moreover, food allergies, prolonged bathing, and tight-fitting clothes have been recognized as additional factors that may exacerbate AD. Therefore, the identification of specific food triggers, limiting bathing time to 5-10 minutes, avoiding irritating fabrics, and the use of loose-fitting garments are advised [18]. In infants, the diaper area is particularly susceptible to skin sensitivity due to excessive moisture, maceration, occlusion, and friction. These conditions lead to an increased skin pH, as fecal ureases break down urea, thereby heightening the activity of fecal enzymes that are highly irritating to the skin [27]. It is recommended to keep the diaper area clean and dry and change the diaper frequently to prevent the risk of diaper dermatitis [28]. Common irritants in children's hygiene products, such as baby wipes, diapers, and certain soaps or lotions, further exacerbate skin sensitivity in this demographic. Children, particularly those engaged in sports, have been reported to develop compromised epidermal barrier function due to exposure to factors such as harmful ultraviolet (UV) rays A and B and physical abrasion from sports activities. These factors necessitate effective photoprotection to mitigate the adverse effects of UV exposure and support the integrity of the epidermal barrier in children [28].

Common pediatric sensitive skin and related barrier-compromise conditions, including AD, contact dermatitis, and diaper dermatitis, affect children across different age groups. These conditions are characterized by chronicity, periods of exacerbation, and complex clinical presentations, making accurate diagnosis and effective management challenging [7,8,29,30]. Patients with sensitive skin conditions, such as AD, are more prone to developing allergic diseases and often experience multimorbidity, where multiple chronic conditions exist. AD is considered the initial clinical manifestation of the "atopic march," which includes allergies, asthma, and food hypersensitivity [31,32]. The clinical burden of AD is substantial in affected children, and they have an increased risk of developing several comorbid conditions. In a population-based study of 216,291 children, 33,591 had a diagnosis of AD, and multimorbidity was present in 43% of them. The most common chronic comorbidities identified were asthma (13.1%), psychosocial disorders (7.9%), and visual impairment (7.8%) [33]. Therefore, the awareness of the range of comorbidities associated with AD in children is crucial, as this could enhance clinical care and assist in developing strategies intended to prevent the progression of AD.

Expert Opinions/Consensus Recommendations

The experts defined sensitive skin as a condition where the skin is more prone to the occurrence of unpleasant sensations (dryness, pruritus, burning, stinging, pain, warmth, and/or erythema) in response to stimuli (physical, chemical, allergenic, and occasionally psychological stress or hormonal) that otherwise should not provoke such sensations, which could be self-reported by older children or based on parental perceptions for younger children (consensus). Sensitive skin may originate from an altered stratum corneum, resulting in an imbalance in the regulation of water homeostasis (consensus). Alterations in epidermal barrier function and neurosensory function play a significant role in the development of sensitive skin. The factors contributing to the alterations in the barrier function of sensitive skin, including AD, are as follows: (1) structural barrier defects such as deficient skin barrier function, abnormal lipid metabolism, and the abnormal formation of epidermal structural proteins such as filaggrin and involucrin; (2) the factors contributing to structural barrier defects in sensitive skin, such as AD, which include (i) imbalance in ceramide composition and levels; (ii) imbalance in the enzyme involved in lipid production that forms the mortar of the skin epidermis, (iii) oxidative stress leading to the production of reactive oxygen species, and (iv) decrease in structural proteins involved in forming the cornified cell envelope and cell bridging; (3) genetic changes such as an imbalance in the expression of genes involved in the production of structural proteins, such as filaggrin and involucrin, keratinocyte differentiation, lipid production, and tight junction formation; (4) microbiome factors, for example, abnormal microbial colonization with pathogenic organisms such as *Staphylococcus aureus* or *Malassezia* sp.; (5) inflammatory factors, for example, the increased production of mediators from inflammatory cells; (6) immunological barrier defects due to the increased skin expression of T-helper type 2 cytokines; and (7) environmental factors, such as pollution, climate change, allergens, and dust.

Sensitive skin syndrome is associated with low skin hydration in infants and children (consensus). In pediatric patients with sensitive skin conditions, such as AD, dryness or scaling is often observed. The experts unanimously reported that symptoms such as itching or pruritus, erythema, rash, and skin dryness are indicative of sensitive skin and related barrier-compromise conditions in pediatric patients.

The experts opined that infants (3-11 months of age) exhibited a high prevalence of sensitive skin in their clinical practice. Primary irritant diaper dermatitis was the most common sensitive skin condition, followed by AD in infants less than 12 months of age. AD was identified as the most common sensitive skin condition observed in children aged >1-3 years, followed by diaper dermatitis (53%) and xerosis (53%). Likewise, in preschool children (aged 3-5 years), AD was identified as the most common sensitive skin condition. Around 46% of experts opined that 5%-19% of their pediatric patients are diagnosed with AD. However, some experts (26%) estimated the prevalence to be between 20% and 39%. Around 66% of experts also mentioned the presence of sensitive skin in patients with xerosis who do not fulfill the criteria for the diagnosis of AD. The experts opined that the prevalence of sensitive skin conditions among pediatric patients has increased over the past decade in India. Lifestyle changes, such as urbanization and environmental pollution, and climate changes, such as rising temperature and humidity levels, are responsible for the rising prevalence of sensitive skin and related barrier-compromise conditions in India. Asthma and allergies are the most common comorbidities observed in pediatric patients with sensitive skin and related barrier-compromise conditions. These are also the most common long-term complications of AD.

Diagnosis of sensitive skin in the pediatric population

Sensitive skin in children primarily presents with objective signs, such as redness, and subjective sensations, including itching, burning, stinging, and tingling. A comprehensive approach, including history and clinical investigation, aids healthcare professionals in achieving an accurate diagnosis [1]. A detailed history specifically regarding sleep patterns, school attendance, and QoL helps provide clues to the diagnosis of sensitive skin. Additionally, self-assessment questionnaires, answered by parents based on objective observations of their child's skin and subjective assessments of their child's behavior, irrespective of the child's age, serve as a valuable tool for identifying patients with sensitive skin [1,34,35]. Moreover, clinical investigations such as the screening of nails, scalp, oral mucosa, and skin provide relevant information for the diagnosis of sensitive skin in children [35]. Skin prick test (SPT) and serological immunoglobulin E (IgE; allergen-specific IgE) testing are beneficial for the diagnosis of hypersensitive reactions (type 1) and immune-mediated allergic reactions [35,36]. According to a study by Boyer et al., a specified symptom sum score system that grades scaliness, roughness, redness, and cracks is another effective clinical tool for evaluating sensitive skin [34]. Furthermore, evaluating a newborn's skin during the initial examination is critical and should include a thorough head-to-toe assessment. The key factors to observe while screening newborn skin include the level of dryness, the presence of scaling, erythema, skin tone, texture, and any physiological skin changes. The neonatal skin condition score (NSCS) is a helpful tool for daily monitoring, assigning scores from 1 to 3 based on the skin's dryness, erythema, and the presence of excoriation or breakdown. A total score of 3 indicates optimal skin condition, while a total score of 9 reflects the most severe issues [28]. TEWL is a major factor in identifying the skin's sensitivity [1,13]. The protective barrier function is weakened when TEWL is high, indicating an increased loss of water from the skin's surface. A compromised barrier increases the skin's vulnerability to environmental stimulants, allergens, and irritants, which increases the risk of sensitive reactions [1,13]. These approaches help dermatologists identify sensitive skin in children and curate personalized treatment plans accordingly.

Expert Opinions/Consensus Recommendations

The lack of specific diagnostic criteria and overlapping symptoms with other dermatological conditions were the main challenges identified in accurately diagnosing sensitive skin and related barrier-compromise conditions in pediatric patients. The experts agreed that in pediatric patients, sensitive skin and related barrier-compromise conditions should be diagnosed based on a comprehensive evaluation, considering both subjective symptoms reported by the child or parent/caregiver and objective clinical signs observed by healthcare providers. The diagnosis of sensitive skin should primarily be made through the observation of objective clinical signs, which include redness, dryness, scaling, and peeling, along with sensations of itching, burning, tightness, stinging, and pain (consensus). The experts highlighted that physical examination and clinical and medical history assessments are the preliminary diagnostic methods most effective for identifying sensitive skin in the pediatric population. Additionally, the experts also emphasized the use of serological (serum IgE) testing and the SPT when type 1 hypersensitivity is suspected in pediatric patients based on examination findings and clinical history. The experts agreed that early identification, facilitating prompt diagnosis and intervention, is perceived as one of the advantages of screening for sensitive skin in infants. A family history of sensitive skin and atopy can help identify children at risk of developing sensitive skin conditions before the onset of clinical signs and symptoms.

Management and prevention of sensitive skin in the pediatric population

Sensitive skin conditions, such as AD, often require multifaceted treatment approaches that include topical medications, frequent moisturizing, avoiding triggers, and managing symptoms such as itching [37]. Emollient therapy is a cornerstone of treatment as it addresses skin barrier dysfunction, which is central to the pathophysiology of AD and other sensitive skin symptoms [15,38]. Emollients play a central role in restoring the skin's barrier integrity, preventing infections, and protecting the skin against further damage [28]. When managing sensitive skin in children, no single emollient works for every child. Healthcare professionals should discuss the types of emollients with the families after understanding the specific circumstances under which the emollient will be used, such as whether it will be applied at home by a parent or by the child at school. The use of sunflower oil, coconut oil, mineral oil, olive oil, and mustard oil has been studied for the care of infant skin [28,39,40]. According to a study by Darmstadt et al., the topical application of sunflower seed oil three times a day was associated with significant improvement in skin condition in preterm neonates [40]. Furthermore, mineral oil, with its effective moisturizing properties due to its emollient and occlusive characteristics, has been found to be a safe option for infants and children. It has limited skin penetration and lacks harmful polyaromatic hydrocarbons, making it a safe choice for infants [28]. For infants and children with conditions such as AD, contact dermatitis, psoriasis, and ichthyosis, selecting the right emollient is key. Petrolatum-based, water-miscible emollients free from preservatives, dyes, and perfumes could be used for preterm or post-term infants, neonates under radiant warmers or phototherapy, and those with sensitive skin conditions (AD, contact dermatitis, psoriasis, and ichthyosis) [28]. Additionally, emollients containing ceramides, lipids, n-palmitoyl ethanolamine, and natural colloidal oatmeal are particularly beneficial for children with AD [28,41].

Colloidal oatmeal has a well-established history of use in dermatology; it is recognized for its natural origin, excellent safety profile, and proven efficacy in managing various conditions, including AD, psoriasis, and drug-induced rashes [42]. The US Food and Drug Administration recognized it as a skin-protectant ingredient in 2003 [43]. Oat lipid extract can increase ceramide levels by 70% in keratinocytes, suggesting enhanced skin barrier function [44]. The extract acts as a potent dual agonist for peroxisome proliferator-‍activated receptor (PPAR) alpha and PPAR beta/delta in primary human keratinocytes, promoting keratinocyte differentiation and boosting ceramide production [44]. Extracts of colloidal oatmeal enhance the expression of the genes involved in epidermal differentiation, tight junction formation, and lipid regulation while providing pH-buffering capabilities [45]. In an in vitro model of AD, colloidal oatmeal significantly increased the expression of several key genes associated with skin barrier function and also protected against barrier damage [45]. Studies show that avenanthramides, isolated from oat grain, inhibit the activity of nuclear factor kappa B and reduce the release of proinflammatory cytokines and histamine, both of which are central to the pathophysiology of inflammatory skin disorders [42]. Topical formulations containing natural colloidal oatmeal can be considered an integral part of the therapeutic approach for AD and other dermatological conditions [42].

The efficacy and safety of colloidal oatmeal and other emollient preparations in xerosis and AD have been investigated in both controlled and uncontrolled studies spanning treatment, maintenance therapy, and prevention, as well as effects on skin barrier and microbiome parameters [46-53]. In a double-blind, randomized, active-controlled trial of 90 children (age: six months to 18 years) with mild-to-moderate AD, an over-the-counter 1% colloidal oatmeal cream was non-inferior to a prescription ceramide-containing barrier cream for change in Eczema Area and Severity Index (EASI) over three weeks, with comparable improvements in investigator-assessed severity and pruritus and a similar tolerability profile [46]. A post hoc analysis of the Black/African American subgroup (N = 49) reported consistent findings [47]. In a study of 61 patients (age: 16-50 years) with mild-to-moderate eczema randomly assigned to 1% colloidal oat cream or a standard, non-fragranced moisturizer, 14 days of treatment with a 1% colloidal oatmeal cream was associated with significant within-group improvement in EASI, AD severity index, skin hydration, barrier function, and skin pH, with trends toward a more favorable microbiome composition [48].

Further, Diluvio et al. showed that colloidal oatmeal effectively and safely reduces itching and irritation associated with AD, as well as alleviates dry skin severity in the pediatric population [49]. These benefits are attributed to the natural components of colloidal oatmeal, which help restore and maintain the skin's barrier function [49]. Liu-Walsh et al. demonstrated that colloidal oatmeal (1%) significantly enhanced the growth, metabolism, and lactic acid production of the skin commensal bacterium *Staphylococcus epidermidis* (*S. epidermidis*) [50]. The study showed that colloidal oatmeal increased the growth rate and metabolic activity of *S. epidermidis* compared with that of *Staphylococcus aureus*, suggesting a potential role in supporting a healthy skin microbiome [50]. This interaction may play a key role in enhancing skin barrier function and promoting microbiome diversity, particularly in skin conditions such as dry and itchy skin and AD [50]. Additionally, Mengeaud et al. showed that a maintenance treatment regimen using a sterile, preservative-free emollient cream containing oat plantlets resulted in a significant reduction in SCORing Atopic Dermatitis (SCORAD) and patient-oriented SCORAD indices (p < 0.001) in children aged six months to six years with moderate AD [51]. The number of flare-ups per subject decreased from 0.76 in the first month to 0.46 in the second month (p < 0.001) and 0.42 in the third month (p < 0.001 versus month 1) [51].

Emollient prophylaxis from birth has been evaluated in large and smaller randomized controlled trials in infants at high familial risk of AD. In the BEEP trial, 1394 infants with a family history of atopic disease received daily emollient (non-oatmeal-based) for the first year or standard care, and there was no reduction in AD incidence [52]. In the STOP-AD trial, 321 newborns received twice-daily application of an oat- and fatty acid-containing emollient from birth to eight weeks or routine skin care; cumulative AD incidence at 12 months was 32.8% versus 46.4% (risk ratio {RR}, 0.71; p = 0.036) in the intention-to-treat analysis. The study demonstrated that the early initiation of daily specialized emollient use (since birth) reduces the incidence of AD in the first year of life in high-risk infants [53]. In another trial of 124 neonates, the daily full-body application of a parent-selected emollient (an oil, a gel, or an ointment) from within three weeks of birth versus no emollient reduced the cumulative incidence of AD at six months (RR, 0.50; 95% confidence interval {CI}, 0.28-0.90; p = 0.017) [54]. It may be important to note that a distinction should be made between the routine use of emollients as part of general skincare in healthy infants and their prophylactic use to prevent AD in infants at high familial risk. The IAP pediatric skin care guidelines recommend emollient application from birth in babies at high familial risk of atopy as a primary prevention measure [28].

Regular emollient use has been shown to reduce the quantity of topical corticosteroid required. In a randomized study of 173 infants (age: <12 months) with moderate-to-severe AD, adding an oat extract-containing emollient for six weeks (162 analyzed) reduced high-potency corticosteroid consumption by 42% (p < 0.05), with no significant reduction in moderate-potency use. SCORAD and quality-of-life scores improved in both groups, indicating that corticosteroid use was reduced without the loss of disease control [55]. In adults with chronic irritant hand eczema, adding a colloidal oatmeal preparation to topical corticosteroid therapy produced greater improvement in clinical severity and quality of life than corticosteroid alone [56]. In a retrospective analysis of UK primary-care records, patients with dry skin and eczema not prescribed emollients had 18% greater odds of receiving a potent or very potent topical corticosteroid and 13% greater odds of receiving a skin-related antimicrobial agent. Among those prescribed emollients, patients never receiving a colloidal oatmeal emollient had ≥21% greater odds of being prescribed a topical corticosteroid or antimicrobial than those prescribed colloidal oatmeal as the first line [57].

Concerns about oat sensitization have been raised, particularly in individuals with preexisting allergies [58]. Further long-term studies are required to determine the potential risk and role of oat-based emollients in causing sensitization. However, these concerns are not consistent. For instance, in the study by Pigatto et al. that assessed the frequency of sensitization to colloidal oat and rice suspensions in 65 children aged six months to two years (43 atopic and 22 nonatopic), none of the children with AD exhibited any allergic reactions to oats during patch testing; mild irritant reactions were seen in five of 43 atopic children [59]. These findings suggest that oat sensitization may be less common in pediatric populations than initially thought.

Optimal skin care in children

Optimal skin care in children requires a consistent and well-informed approach to daily emollient use and photoprotection. Emollients should be applied within 3-5 minutes after a brief bath (5-10 minutes) in lukewarm water, with the skin gently patted dry. The frequency of application should be every 4-6 hours, depending on the degree of dryness, to maintain hydration and protect the skin barrier [28]. Barriers to treatment adherence by patients and caregivers include misunderstanding instructions, forgetting medication, disliking the formulation or side effects, and the fear of adverse effects [37,60]. Another common challenge involves skin irritation or sensitization caused by emollient additives. Some emollients contain preservatives, fragrances, or other ingredients that can irritate sensitive skin or even trigger allergic reactions and should be avoided [17]. Effective patient engagement, dedicated consultation time, and thorough education of patients and caregivers are essential for addressing these issues and enhancing treatment adherence rates [60]. The American Academy of Pediatrics (AAP) also advises minimizing sun exposure for all children between 10:00 am and 4:00 pm [28,61,62]. Further, the AAP recommends the use of protective clothing, wide-brimmed hats, and UV-protective sunglasses, in addition to the application of broad-spectrum sunscreen with a sun protection factor of 15 or higher for infants older than six months and young children [28,61,62]. These measures ensure comprehensive protection from UV radiation while minimizing the risk of skin irritation or allergic reactions [28,61,62]. In India, the routine use of sunscreens is not yet widely adopted across the general population. For children under 12 years of age, certain sunscreen formulations such as liquids, sprays, and alcohol-based gels are more likely to cause skin irritation and should be avoided [28]. In summary, maintaining the skin barrier in children with sensitive skin relies on consistent emollient use, the careful selection of nonirritating formulations, thorough caregiver education, and appropriate sun protection.

Expert Opinions/Consensus Recommendations

The experts highlighted that parents of babies/children with sensitive skin are usually concerned about bathing practices (e.g., frequency, the temperature of the water, and the duration of the bath), postbathing care (e.g., the time of emollient application after the bath, type and quantity of emollient, method of emollient application, and type of clothing), the time of the resolution of signs and symptoms, and the usage of steroids. The most common challenge faced by patients with AD is the discontinuation of the use of emollients once the symptoms subside, followed by skin irritation or sensitization caused by additives in emollients. The experts pointed out that there was a lack of awareness among parents and/or caregivers regarding the long-term nature of treatment and the need for ongoing maintenance to effectively manage the condition. Maintaining a consistent skincare routine could be difficult, especially when trying to incorporate it into a child's busy daily activities. These factors can lead to gaps in treatment adherence, potentially prolonging or worsening skin conditions.

The experts opined that when choosing an emollient for children with sensitive skin, the following factors must be considered: (1) It should be hypoallergenic, including thorough fragrance evaluation for allergenicity and irritant potential, and noncomedogenic (specific to the face). (2) Formulations must maintain the pH balance of the skin. (3) It should have minimal ingredients that offer optimal therapeutic benefits while providing immediate and long-term relief from symptoms.

The experts opined that managing sensitive skin conditions in pediatric patients can effectively limit the severity and progression of the condition and improve QoL for both the child and the parent/caregiver. The cessation of rubbing and scratching serves as a sign for assessing immediate relief in children with sensitive skin and related barrier-compromise conditions. For evaluating the enhancement in QoL in children, 93% of experts concurred that improvement in sleep was a key parameter, while 80% of experts reported that the relief and reduction of symptoms, such as itching, are important factors. Most experts (93%) acknowledged that the antipruritic action of emollients with skin hydration would provide good relief of symptoms. The experts opined that emollients for sensitive skin and related barrier-compromise conditions should (i) retain hydration while repairing the skin barrier; (ii) activate the endogenous production of ceramides to help restore the skin's natural ceramide balance; (iii) decrease TEWL by helping to develop a hydrolipid barrier on the skin; (iv) restore the skin microbiome balance; (v) provide immediate and long-lasting relief from itching, redness, and irritation; and (vi) moisturize and protect the skin from irritation.

Emollients with additives that irritate or sensitize the skin should be avoided. Around 57% of experts recommended the initiation of consistent emollient usage in babies from birth. However, some experts suggested that the decision should depend on age factors and clinical signs, such as the redness and dryness of the skin. For pediatric patients with AD, it is advisable to bathe in lukewarm water for 5-10 minutes, use a moisturizing cleanser, gently pat the skin dry, and then apply emollients liberally (consensus). The experts agreed that emollients should be applied to damp skin soon after bathing for maximum benefits (i.e., better penetration). Recommendations for applying emollients for managing AD in children typically involve twice-daily use, but this frequency can differ based on individual and environmental factors, as well as the degree of skin dryness (consensus). The experts opined that colloidal oatmeal-based emollients are an ideal therapeutic option for managing sensitive skin in the pediatric population due to their (i) ability to restore the skin's natural barrier and enhance endogenous ceramide, (ii) ability to restore skin microbiome balance and skin homeostasis, (iii) natural anti-inflammatory properties that soothe irritation, (iv) antipruritic properties that reduce itching and discomfort, (v) prebiotic properties, (vi) ability to limit AD progression, (vii) ability to balance the skin's pH levels, (viii) high lipid content that moisturizes and prevents water loss, (ix) antioxidants that protect the skin from environmental damage, and (x) gentle composition suitable for all skin types, including sensitive and eczema-prone skin.

Applying a colloidal oatmeal-based emollient twice daily, combined with a colloidal oatmeal-based cleanser to bathe babies and young children with AD, can significantly improve symptoms, such as itching, dryness, and roughness, and overall disease severity (consensus). Furthermore, the early initiation and long-term usage of colloidal oatmeal-based emollients help in the reduction of flare severity and frequency and reliance on corticosteroids and antimicrobials. Around 60% of experts highlighted that colloidal oatmeal functions as a skin prebiotic and contributes to restoring skin microbiome diversity in various skin conditions, including dry/itchy skin and AD, in infants and children. The expert panel recommends colloidal oatmeal-based emollients in xerosis, AD, and allergic contact dermatitis due to their prebiotic properties and soothing effects and to activate endogenous ceramide production, which helps restore the skin's barrier and integrity. It should be emphasized, however, that in allergic contact dermatitis, the cornerstone of management remains the identification and avoidance of the causative allergen.

Strengths and limitations of the consensus process

Strengths

This modified Delphi study offers a structured synthesis of expert opinion on a clinically important but under-addressed topic in Indian pediatric practice. Its strengths include the use of a structured, two-round modified Delphi approach with an interim discussion, the participation of a multidisciplinary panel of pediatricians and dermatologists drawn from across the major regions of India, and anonymized independent voting intended to encourage candid, unbiased feedback. The panel was assembled purposively on the basis of documented clinical and academic expertise in pediatric skincare; this purposive approach was a pragmatic choice appropriate to a consensus exercise.

Limitations

This consensus process has several limitations that should be considered when interpreting the recommendations. First, the consensus process did not include patient, caregiver, nursing, primary-care, or public-health perspectives. Second, consensus was defined primarily on the basis of the mean Likert score. Although the number and proportion of experts agreeing with each statement are also reported (Table 2), a mean-based threshold may mask differing opinions. Third, the quality of the supporting evidence and the strength of individual recommendations were not formally graded using an established evidence appraisal framework. Consequently, the recommendations should be interpreted as expert consensus informed by the available evidence rather than as formally graded, evidence-based guidelines. Fourth, one panelist did not participate in the second round of voting. This affected only the subset of statements revoted in that round, for which the reported proportions are based on 14 respondents. Finally, the consensus was commercially funded, and panelists received honoraria for their participation, which may introduce potential for sponsor- or product-related influence. To minimize this risk, clinical panelists independently drafted, discussed, and voted on all statements, while sponsor-affiliated authors did not vote or participate in the clinical deliberations.

## Conclusions

Sensitive skin and related barrier-compromise conditions are a prevalent concern in the pediatric population, particularly in India, where environmental factors and genetic predisposition significantly influence their occurrence. According to experts, early interventions, such as using gentle, pH-balanced cleansers and moisturizers, are crucial for maintaining skin barrier function. Additionally, the experts highlighted that maintaining a consistent skin care regimen can be challenging because of a child's busy schedule, which may lead to gaps in treatment adherence, potentially prolonging or worsening skin conditions.

Furthermore, the experts also highlighted that the use of colloidal oatmeal has emerged as a promising ingredient in managing sensitive skin, as it possesses skin-soothing properties that may reduce inflammation and enhance the skin barrier, and may help reduce reliance on corticosteroids. Addressing the unique challenges posed by India's diverse climate and socioeconomic factors, these consensus-based recommendations may help inform the future development of evidence-based guidelines tailored to the Indian pediatric population. These consensus-based recommendations are intended to complement, rather than replace, evidence derived from high-quality prospective clinical studies in the Indian pediatric population. Ultimately, an integrative approach that includes early diagnosis, patient education, individualized care, and collaboration among healthcare providers is essential to optimize outcomes, reduce the burden on both children and their families, and improve QoL.

## Appendices

Table 3 shows the composition of the panel.

**Table 3:** Demographic and professional characteristics of the Delphi expert panel Source: original table developed by the authors. Zone denotes the region of India in which the panelist practices

Panelist number	Gender	Zone	Geographic location	Specialty	Type of practice
1	Male	North	Delhi	Pediatrics	Private
2	Male	South	Chennai	Dermatology	Government
3	Male	South	Bangalore	Pediatrics	Private
4	Male	South	Chennai	Pediatrics	Private
5	Male	South	Hyderabad	Pediatrics	Private
6	Male	West	Mumbai	Pediatrics	Private
7	Male	West	Mumbai	Pediatrics	Private
8	Male	North	Jaipur	Pediatrics	Private
9	Male	West	Mumbai	Pediatrics	Private
10	Male	South	Chennai	Pediatrics	Government
11	Male	East	Durgapur	Pediatrics	Private
12	Female	North	New Delhi	Dermatology	Private
13	Female	West	Mumbai	Dermatology	Mixed
14	Male	South	Chennai	Dermatology	Private

Table 4 shows the questionnaire.

**Table 4:** Questionnaire

Question/statement			Options								
Does the following definition encapsulate the condition of sensitive skin? Sensitive skin is "a syndrome defined by the occurrence of unpleasant sensations (stinging, burning, pain, pruritus, and tingling sensations) in response to stimuli that normally should not provoke such sensations, which could be self-reported by older children and based on parental perceptions for younger children." Stimuli refer to the multiple factors, which may be physical (ultraviolet {UV}, heat, cold, and wind), chemical (cosmetics, soaps, water, and pollutants), genetic, and occasionally psychological (stress) or hormonal.	Strongly agree	Agree				Neutral		Disagree			Strongly disagree
Based on their physiological parameters, the spectrum of sensitive skin can be classified into three different types: type I, low epidermal barrier function group; type II, inflammation group with normal barrier function and inflammatory changes; and type III, pseudo-healthy group in terms of normal barrier function and no inflammatory changes. In your expert opinion, how valid do you find the classification of sensitive skin?	Valid, that is, the classification accurately reflects the diverse manifestations of sensitive skin			Moderately valid. While the classification provides insights, it may not fully capture all nuances of sensitive skin			Not valid. The classification oversimplifies the complexity of sensitive skin, neglecting important variations			Uncertain/no opinion	
In your clinical practice, how would you rate the perceived prevalence of sensitive skin and conditions including atopic dermatitis among pediatric patients?	Less than 5%	5%-19%				20%-39%		40%-59%			More than 60%
In your expert opinion, what are the most common sensitive skin conditions observed in pediatric patients? Please select all that apply.	Infancy (less than 12 months)	Atopic dermatitis			Diaper dermatitis		Seborrheic dermatitis		Xerosis		Others (please specify)
	Age of >1 year to three years	Atopic dermatitis			Diaper dermatitis		Seborrheic dermatitis		Xerosis		Others (please specify)
	Preschool (3-5 years)	Atopic dermatitis			Impetigo		Rosacea		Psoriasis		Perioral and periorbital dermatitis
	Contact dermatitis	Seborrheic dermatitis			Xerosis		Others (please specify)				
In your practice, how would you rate the clinician-perceived prevalence of atopic dermatitis among pediatric patients?	Less than 5%	5%-19%				20%-39%		40%-59%			More than 60%
In your clinical practice, how would you rate the perceived prevalence of xerosis among pediatric patients?	Less than 5%	5%-19%				20%-39%		40%-59%			More than 60%
In your clinical practice, which demographic group exhibits a higher prevalence of sensitive skin?	Age	Newborn (0-2 months)			Infants (3-11 months)		Toddlers (1-3 years)		Preschool (3-6 years)		School age (6-12 years)
	Adolescents (12-16 years)	Gender			Girls		Boys				
At what age do patients typically first present with symptoms of sensitive skin and conditions including atopic dermatitis in your clinical practice?	0-2 months	3-11 months			1-3 years		3-6 years		6-12 years		12-16 years
Do you believe that the prevalence of sensitive skin conditions among pediatric patients has increased over the past decade in India?	Yes						No				
If yes, what are the reasons for the rising prevalence of sensitive skin and conditions including atopic dermatitis in India?	Lifestyle changes such as urbanization	Environmental pollution				Climate change such as rising temperature and humidity levels		Excessive use of harsh soaps and detergents			Inadequate skincare practices
What is your expert opinion on the below statement? The prevalence of sensitive skin tends to decrease with advancing age.	Strongly agree	Agree				Neutral		Disagree			Strongly disagree
Sensitive skin and conditions including atopic dermatitis in pediatric patients often have a substantial impact on their quality of life, such as their mood and ability to go back to sleep.	Strongly agree	Agree				Neutral		Disagree			Strongly disagree
In your expert opinion, which of the following mechanisms do you believe plays a significant role in the development of sensitive skin?	Alterations of epidermal barrier function			Neurosensory function			Both			Others (please specify)	
What are the factors that contribute to the alterations in the barrier function of sensitive skin?	Genetic changes such as imbalance in expression of genes involved in the production of structural proteins such as filaggrin and involucrin, involved in keratinocyte differentiation, lipid production, and tight junction formation	Environmental factors such as pollution, climate change, allergens, and dust			Imbalance of skin microbiome		Structural barrier defects		Immunological barrier defects due to increased skin expression of Th2 cytokines		Others (please specify)
What are the factors that contribute to the structural barrier defects in sensitive skin?	Imbalance in ceramide composition and levels	Imbalance in enzymes involved in lipid production, which forms the mortar of the skin epidermis				Oxidative stress leading to the production of reactive oxygen species		Decrease in structural proteins forming the cornified cell envelope and cell bridging			Others (please specify)
Do you agree that sensitive skin may originate from altered stratum corneum, resulting in an imbalance in the regulation of water homeostasis in sensitive skin?	Strongly agree	Agree				Neutral		Disagree			Strongly disagree
Apart from a strong genetic influence and environmental factors, are the following characteristic features in the pathophysiology of atopic eczema/atopic dermatitis?	Immunological factors. Immune deviation toward the T-helper (Th2) pathway with increased IgE production	Inflammatory factors. Increased production of mediators from inflammatory cells				Skin Barrier components. Deficient skin barrier function, abnormal lipid metabolism, and abnormal formation of epidermal structural proteins such as filaggrin and involucrin		Microbiome factors. Abnormal microbial colonization with pathogenic organisms such as Staphylococcus aureus or Malassezia sp.			Strong psychosomatic influence
Considering the studies that have shown only a fraction (few to 50%) of patients carry FLG mutations and 60% of these carriers do not develop AD, do you believe that filaggrin mutation is "not" the only major cause of sensitive skin and conditions such as atopic dermatitis?	Strongly agree	Agree				Neutral		Disagree			Strongly disagree
Sensitive skin syndrome is associated with lower skin hydration in infants and children.	Strongly agree	Agree				Neutral		Disagree			Strongly disagree
In your expert opinion, what are the main challenges in accurately diagnosing sensitive skin and conditions including atopic dermatitis in pediatric patients?	Difficulty in accurate reporting by parents	Lack of clearly visible manifestations				Lack of specific diagnostic criteria		Overlapping symptoms with other dermatological conditions			Others (please specify)
In your expert opinion, what are the main challenges faced by pediatric patients and their caregivers in adhering to treatment plans for sensitive skin and conditions including atopic dermatitis?	Difficulty in applying topical medications (e.g., emollients and ointments)	Cost of prescribed treatments			Challenges in maintaining a consistent skincare routine amidst the child's daily activities		Concerns about potential side effects of medications		Lack of understanding about the importance of treatment adherence		Lack of awareness of the duration of treatment and maintenance required for effectively managing sensitive skin conditions
	Others (please specify)										
Do you agree that the level of awareness among caregivers and parents regarding sensitive skin conditions in pediatric patients is progressively increasing?	Strongly agree	Agree				Neutral		Disagree			Strongly disagree
How can we best identify children at risk for the development of sensitive skin conditions before the onset of clinical signs and symptoms?	Atopic parents			Family history of sensitive skin			Siblings with allergies			Others (please specify)	
What is typically the first symptom or sign observed in pediatric patients with sensitive skin and conditions including atopic dermatitis?	Itching or pruritus	Redness or erythema			Dryness or scaling		Rash or dermatitis		Pain or discomfort		Tingling sensation
	Others (please specify)										
Which of the following symptoms do you consider to be indicative of sensitive skin and conditions including atopic dermatitis in pediatric patients?	Itching or pruritus	Redness or erythema			Dryness or scaling		Rash or dermatitis		Pain or discomfort		Tingling sensation
	Others (please specify)										
In your expert opinion, do pediatric populations with sensitive skin exhibit more clinical signs of skin dryness compared to those without sensitive skin?	Strongly agree	Agree				Neutral		Disagree			Strongly disagree
Does sensitive skin represent a skin condition or manifestations of other disorders?	Yes						No				
Do you agree that sensitive skin can serve as a cutaneous manifestation of underlying skin disorders?	Strongly agree	Agree				Neutral		Disagree			Strongly disagree
Which of the following cutaneous and extracutaneous disorders do you commonly associate with sensitive skin symptoms? Please select all that apply.	Atopic dermatitis	Psoriasis				Rosacea		Adverse reactions to skin care products			Others (please specify)
In your expert opinion, do pediatric patients with sensitive skin conditions experience coexisting health issues or comorbidities?	Yes						No				
What are the most common comorbidities observed in pediatric patients with sensitive skin and conditions including atopic dermatitis? Please select all that apply.	Asthma	Allergies			Obesity/overweight		Anemia		Infections		Others (please specify)
Do you agree that a history of childhood atopic dermatitis is more frequent in children with sensitive skin and that children with sensitive skin often report a concomitant atopic condition?	Strongly agree	Agree				Neutral		Disagree			Strongly disagree
What are the most common long-term complications of atopic dermatitis?	Cutaneous infections	Scarring, hyperpigmentation, or hypopigmentation			Asthma and allergies		Eye problems		Mental health		Sleep problems
	Cardiovascular problems										
Do you agree that filaggrin mutation and reduction in filaggrin levels are not the only cause of atopic dermatitis, but additional factors in the development of AD include environmental factors, other genetic predisposition, biochemical changes, and immune dysfunction?	Strongly agree	Agree				Neutral		Disagree			Strongly disagree
What are the perceived benefits of screening for sensitive skin and atopic skin in infants?	Early identification leading to prompt diagnosis and intervention	Improved management and treatment outcomes			Prevention of secondary infections and comorbidities		Enhanced quality of life for infants and caregivers		Long-term health benefits such as the prevention of chronic skin conditions or atopic march		Others
Allergen sensitization through a defective skin barrier can lead to clinical diseases or predispose to the development of atopic dermatitis (AD) and food allergy. (Please select one of the below options.)	Strongly agree	Agree				Neutral		Disagree			Strongly disagree
According to you, parents of babies/children with sensitive skin would be concerned about the following:	Bathing practices. Frequency, the temperature of water, and the duration of the bath	Postbathing care. The time of emollient application postbath, type and quantity of emollient, method of application of emollient, and type of clothing			Risk of food allergy		Can diapers be used, where applicable		Usage of steroids		Weather dependency of symptoms and signs
	Time of the resolution of signs and symptoms										
Sensitive skin and conditions including atopic dermatitis in pediatric patients should be diagnosed based on a comprehensive evaluation, considering both subjective symptoms reported by the child or parent/caregiver and objective clinical signs observed by healthcare providers.	Strongly agree	Agree				Neutral		Disagree			Strongly disagree
In your clinical practice, do you diagnose sensitive skin primarily through the observation of objective clinical signs, which include redness, dryness, scaling, and peeling, along with sensations of burning, tightness, itching, stinging, and pain?	Strongly agree	Agree				Neutral		Disagree			Strongly disagree
Based on your expertise, which preliminary diagnostic methods are most effective for identifying sensitive skin in the pediatric population?	Clinical and medical history	Physical examination				Elimination diets (for suspected food allergies)		Observing the skin's reaction to specific skin care products or ingredients			Others
In your clinical practice, do you employ any advanced diagnostic techniques for identifying sensitive skin in pediatric patients? Please select your response and specify any methods used.	Yes						No				
Based on your expertise, which advanced diagnostic methods are most effective for identifying sensitive skin in the pediatric population?	Skin barrier function tests	Skin biopsy				Skin prick testing		Blood tests			Others
In your experience, which allergy panel tests have proven most effective in identifying specific allergens or triggers that contribute to skin sensitivity in pediatric patients?	Prick test	Serological (serum IgE) testing				Patch test		Basophil activation test (BAT)			Others
Do you use any skin barrier function tests for diagnosing sensitive skin in pediatric patients?	Yes						No				
If you have answered "yes" to the previous question, which are the skin barrier function tests commonly used in your clinical practice for diagnosing sensitive skin among pediatric patients?	Transepidermal water loss (TEWL) measurement	Instrumental measurement of stratum corneum hydration				Lipid profile analysis		pH measurement			Others
In your clinical practice, which in-office tests do you frequently order for diagnosing sensitive skin and conditions including AD among pediatric patients?	Lactic acid sting test	Chloroform menthol test			Sensory perception threshold management test		Irritant reactivity test		Others		I do not order any in-office tests
Do you use any scoring methods or scales for the diagnosis of atopic dermatitis (AD) among pediatric patients in your daily practice?	Yes						No				
If you indicated "yes" in response to the previous question, could you identify from the list below which diagnostic method(s) you commonly use for diagnosing AD in pediatric patients?	SCORing Atopic Dermatitis (SCORAD)	Williams criteria				Eczema Area and Severity Index (EASI)		Hanifin and Rajka diagnostic criteria			Others
What is the objective of sensitive skin condition management in pediatric patients?	Immediate relief of symptoms	Limit the severity and progression of the condition			Reduction in flare frequency and prolonging the interval between flares		Improvement in QoL for the child and parent		Repair and maintenance of skin barrier		Others
Do you agree that moisturizers/emollients play a crucial role in the daily management of individuals with sensitive skin?	Strongly agree	Agree				Neutral		Disagree			Strongly disagree
Which of the following factors do you consider in patients while choosing an appropriate emollient for children with sensitive skin including AD?	Skin type	Degree of dryness			Age of the patient		Site of application		Immediate and long-term relief of symptoms		Reduction in frequency and prolonging interval between flares
	Others										
In your clinical practice, how do you assess immediate relief of signs and symptoms in babies with sensitive skin and conditions including AD?	Child stops crying			Reduction in redness			Child stops/reduces rubbing and itching			Others	
Which of the following factors do you consider for improvement in quality of life (QoL) in children?	Improvement in sleep			Relief of symptoms and signs such as itchiness			Reduction in the severity of symptoms			Others	
What are the most important factors to consider when choosing an emollient for children with sensitive skin?	Emollients with minimal ingredients that offer optimal therapeutic benefits	Emollients with multiple ingredients that offer optimal therapeutic benefits			Emollients with the right natural ingredients, plant-based components, that are demonstrated to be effective while being well-tolerated and are approved by regulatory bodies		Emollients formulated to maintain skin pH balance		Absorbed quickly, providing optimal hydration		Emollients providing immediate and long-term relief of symptoms
	Hypoallergenic, fragrance-free, and noncomedogenic										
In your expert opinion, what are the key desired characteristics of emollient therapy for sensitive skin conditions in babies including AD?	Restores hydration and repairs the skin barrier	Activates the endogenous production of ceramides to help restore the skin's natural ceramide balance			Decreases transepidermal water loss by helping develop a hydrolipid barrier on skin		Recognized by international regulatory bodies/authorities as a skin-protectant ingredient that moisturizes and protects the skin from irritation		Does not contain additives that irritate or sensitize the skin		Has anti-inflammatory and antioxidant properties
	Provides immediate and long-lasting relief from itching, redness, and irritation	Does not clog pores			Restores skin microbiome balance		Maintains skin pH balance		Others		
Which of the following functions, aside from skin hydration, do you recognize as benefits provided by emollients in your clinical practice?	Anti-inflammation and antioxidant	Antipruritic			Antimitotic		Balances skin microbiota and maintains skin homeostasis		Anti-allergic		Limits progression of atopic march
	Others										
The right emollient can not only restore but also repair the skin barrier by the stimulation of endogenous ceramide production.	Strongly agree	Agree				Neutral		Disagree			Strongly disagree
While the exogenous fortification of ceramides will only help to replenish the depleted ceramides, the activation of endogenous ceramide production will help to restore the natural ceramide balance of the skin.	Strongly agree	Agree				Neutral		Disagree			Strongly disagree
Emollients used in the management of sensitive skin in the pediatric population should be free of potentially irritating substances including thorough fragrance evaluation for allergenicity and irritant potential.	Strongly agree	Agree				Neutral		Disagree			Strongly disagree
Considering that all emollients are not equal as different types of emollients exhibit diverse physiological effects on the skin barrier in subjects with sensitive skin and conditions including atopic dermatitis, do you believe that the written recommendation of emollients should be preferred over oral recommendation?	Strongly agree	Agree				Neutral		Disagree			Strongly disagree
Skin rehydration and skin barrier repair with regular and long-term usage of emollients reduce the need for other topical treatments.	Strongly agree	Agree				Neutral		Disagree			Strongly disagree
The early and regular use of emollients that stimulate/activate the endogenous production of ceramides to help restore the skin's natural ceramide balance in infants and children with sensitive skin and conditions including AD extends the duration between flares and reduces the intensity and frequency of flares, irrespective of severity of the condition.	Strongly agree	Agree				Neutral		Disagree			Strongly disagree
Open-ended: Based on your experience, which formulation of moisturizers (creams, ointment, lotion, or gel) do you find to be most user-friendly and effective in enhancing treatment adherence among pediatric patients with sensitive skin? Also, explain why.											
As emollients could prevent atopic march and prevent the development of sensitive skin conditions including AD, when would you recommend the initiation of regular emollient usage in babies?	From birth	From three months onward				From six months onward		Age-independent and dependent on appearance of signs such as dryness and redness			Others
Emollients should be applied to damp skin soon after bathing for maximum benefits (i.e., better penetration). (Please select one option from the options below.) Do you agree that the management of AD should not adopt a "one-size-fits-all" approach and treatments should be tailored to individual pathogenic reactions and reactivity patterns?	Strongly agree	Agree			Neutral		Disagree		Strongly disagree		Strongly agree
	Agree	Neutral			Disagree		Strongly disagree				
Do you agree that for pediatric patients with AD, it is advisable to bathe in lukewarm water for 5-10 minutes, use a moisturizing cleanser, gently pat the skin dry, and then apply emollients liberally?	Strongly agree	Agree				Neutral		Disagree			Strongly disagree
The early initiation of emollient usage is recommended from day 1 (day 1 of diagnosis OR day 1 of appearance of symptoms or increase in severity OR day 1 in all high-risk infants OR day 1 in all babies) for the management of sensitive skin conditions, since they are considered the cornerstone of the management paradigm. In your clinical practice, do you recommend the initiation of regular emollient usage for improving signs and symptoms of sensitive skin including AD symptoms and tolerability in children, including infants?	Strongly agree	Agree				Neutral		Disagree			Strongly disagree
Recommendation for applying emollients in managing AD in children typically involves twice-daily use, but this frequency can differ based on individual and environmental factors, as well as the degree of skin dryness?	Strongly agree	Agree				Neutral		Disagree			Strongly disagree
What are the most common challenges your patients with AD face when it comes to regularly using moisturizers?	Discontinue using moisturizer once the symptoms subside	Not satisfied with the type of formulation			Patients worried about the ingredients		Moisturizer contains additives that irritate or sensitize the skin		Allergic side effects reported		Others
Would you recommend an emollient that, in addition to skin hydration, plays a role in restoring the skin barrier integrity by upregulating the enzymes and genes downregulated in AD/sensitive skin?	Strongly agree	Agree				Neutral		Disagree			Strongly disagree
To what extent do you agree with the following statement? "Effective photoprotection in babies/children using an ideal sunscreen plays a pivotal role in safeguarding the skin against sensitivity, sunburns, and the exacerbation of underlying skin conditions."	Strongly agree	Agree			Neutral		Disagree		Strongly disagree		Strongly agree
The regular usage of colloidal oatmeal-based emollients has the potential to prevent allergen penetration/sensitization and subsequently prevent progression of the condition (which may include progression of atopic march).	Agree	Neutral			Disagree		Strongly disagree				
Colloidal oatmeal-based moisturizers should be recommended for pediatric patients with sensitive skin due to their multitherapeutic benefits such as ability to stimulate/activate endogenous ceramide production, their prebiotic properties, and overall soothing effects, which help in restoring skin integrity.	Strongly agree	Agree				Neutral		Disagree			Strongly disagree
Colloidal oatmeal-based emollients can be used as early as the first week of life to prevent AD to maintain skin barrier integrity and reduce the risk of sensitive skin conditions, especially in high-risk infants.	Strongly agree	Agree				Neutral		Disagree			Strongly disagree
Regular prophylactic application of colloidal oatmeal-based emollients initiated in early infancy among high-risk infants prevents the occurrence of AD.	Strongly agree	Agree				Neutral		Disagree			Strongly disagree
In your expert opinion, what makes oatmeal-based emollients an ideal therapeutic option for managing sensitive skin in the pediatric population?	Natural anti-inflammatory properties that soothe irritation	Ability to restore the skin's natural barrier			Limits AD progression		Enhance endogenous ceramide		The presence of antioxidants that protect skin from environmental damage		High lipid content that moisturizes and prevents water loss
	Gentle composition suitable for all skin types, including sensitive and eczema-prone skin	Antipruritic properties that reduce itching and discomfort			Ability to balance the skin's pH levels		Prebiotic properties		Restores skin microbiome balance and skin homeostasis		Others
Colloidal oatmeal-based emollients can nourish and protect a baby's delicate and fragile skin and limit the progression of AD.	Strongly agree	Agree				Neutral		Disagree			Strongly disagree
Colloidal oatmeal restores and repairs the skin barrier by not only forming a protective hydrolipid film on the skin but also aiding in water-binding and moisture-retention properties of the SC.	Strongly agree	Agree				Neutral		Disagree			Strongly disagree
Colloidal oatmeal-based emollient is clinically effective in managing sensitive skin including atopic skin conditions in children and is well-tolerated.	Strongly agree	Agree				Neutral		Disagree			Strongly disagree
Colloidal oat-based emollient should be recommended as part of core management from day 1 in sensitive skin and conditions including atopic skin, to moisturize the skin, repair the skin barrier, and help relieve symptoms.	Strongly agree	Agree				Neutral		Disagree			Strongly disagree
Initial treatment strategy for sensitive skin and conditions including AD in children should involve applying a colloidal oatmeal-based barrier repair emollient along with or without standard-of-care treatment to restore and repair the skin barrier while maintaining natural skin homeostasis.	Strongly agree	Agree				Neutral		Disagree			Strongly disagree
Do you agree that skin rehydration and repair with colloidal oatmeal-based emollients potentially reduce the requirement of topical steroids and other anti-inflammatory agents?	Strongly agree	Agree				Neutral		Disagree			Strongly disagree
Routine application of colloidal oatmeal-based emollients on infants can alleviate signs of irritation and discomfort associated with dryness.	Strongly agree	Agree				Neutral		Disagree			Strongly disagree
Colloidal oatmeal functions as a skin prebiotic and contributes to the restoration of skin microbiome diversity in various skin conditions, including dry/itchy skin and AD in infants and children.	Yes				No				Maybe		
Applying a colloidal oatmeal emollient twice daily, combined with using a colloidal oatmeal cleanser for bathing babies and young children with AD, can significantly improve symptoms such as itching, dryness, roughness, and overall disease severity.	Strongly agree	Agree				Neutral		Disagree			Strongly disagree
Colloidal oatmeal-based emollients can exert multitherapeutic beneficial effects on the skin barrier through the enhancement of barrier homeostasis and are thus a total package for the management of sensitive skin conditions including AD.	Strongly agree	Agree				Neutral		Disagree			Strongly disagree
The early initiation and long-term usage of colloidal oatmeal-based emollients helps in the following:	Reduction in the severity and frequency of flares	Reduction in the usage of topical anti-inflammatory agents including topical corticosteroids and antimicrobials			Reduction in the severity of the condition		Limit or arrest the progression of the condition		Prevention of signs and symptoms of sensitive skin conditions including AD		Building a foundation for a lifetime of healthy skin
	All of the above										
Colloidal oatmeal-based emollients can be preferred skincare products for the management of xerosis due to the following benefits offered:	Re-establish skin hydration and lipids	Decrease inflammation			Enhance the barrier activity		Exert a soothing effect		Limit or arrest the progression of the condition		All of the above
Per your clinical practice and available evidence, the regular usage of colloidal oatmeal-based emollients could reduce the severity of sensitive skin conditions including AD over time by limiting disease progression in children.	Strongly agree	Agree				Neutral		Disagree			Strongly disagree
Colloidal oatmeal-based emollient therapy helps in reducing the clinical manifestations of AD, including dryness, pruritus, erythema, scaling, and roughness in neonates, infants, and children.	Strongly agree	Agree			Neutral		Disagree		Strongly disagree		Strongly agree
Colloidal oatmeal-based moisturizers should be the cornerstone for the effective management of sensitive skin and conditions including atopic dermatitis and can be recommended in children with xerosis, rosacea, atopic dermatitis, damage from topical steroids, seborrheic dermatitis, allergic contact dermatitis, psoriasis, perioral dermatitis, and photosensitive dermatoses.	Agree	Neutral			Disagree		Strongly disagree				
Colloidal oatmeal-based emollients can significantly reduce the intensity of pruritus in children with sensitive skin and conditions including AD.	Strongly agree	Agree				Neutral		Disagree			Strongly disagree
Colloidal oatmeal-based emollients can help in breaking the itch-scratch cycle due to the presence of avenanthramides, which inhibit neurogenic inflammation, NF-kB pathway, and histamine signaling.	Strongly agree	Agree				Neutral		Disagree			Strongly disagree
Colloidal oatmeal-based emollients can simulate not only the production of filaggrin but also other key skin barrier biomarkers, genes involved in keratinocyte differentiation, lipid production, and tight junction formation.	Strongly agree	Agree				Neutral		Disagree			Strongly disagree
Factors that parents should be made aware of for the prevention and management of sensitive skin conditions including atopic dermatitis in babies/children.	Bathing practices	Moisturizing practices			Clothing recommendations		The use of sunscreen for skincare in babies/children		Common irritants that could trigger atopic skin conditions		Climate and environmental factors that could act as triggers
	Daily care and prevention practices	Others									
Modified set of questions (after the meeting)											
Sensitive skin is defined as "a condition where the skin is more prone to the occurrence of unpleasant sensations (such as dryness, pruritus, burning, stinging, pain, warmth, and erythema) in response to stimuli that otherwise should not provoke such sensations, which could be self-reported by older children or based on parental perceptions for younger children." Stimuli refer to multiple factors, which may be physical, chemical, allergens, and occasionally psychological (stress) or hormonal.	Strongly agree	Agree				Neutral		Disagree			Strongly disagree
What are the reasons for the rising prevalence of sensitive skin conditions including atopic dermatitis in India?	Lifestyle changes such as urbanization	Environmental pollution			Climate change such as rising temperature and humidity levels		Excessive use of harsh soaps and detergents		Inadequate skincare practices		All of the above
The prevalence of sensitive skin tends to diminish with increasing age?	Strongly agree	Agree				Neutral		Disagree			Strongly disagree
Do you agree that the level of awareness among parents and caregivers regarding sensitive skin in the pediatric age group is progressively increasing/better compared to yesteryears?	Strongly agree	Agree				Neutral		Disagree			Strongly disagree
Which of the following cutaneous and extracutaneous disorders do you commonly associate with sensitive skin symptoms in the pediatric age group?	Atopic dermatitis	Psoriasis				Rosacea		Adverse reactions to skin care products			Others (please specify)
What are the most common long-term complications of AD?	Cutaneous infections	Hyperpigmentation or hypopigmentation			Asthma and allergies		Eye problems		Adverse effects on quality of life		Cardiovascular problems
According to you, parents of babies/children with sensitive skin are usually concerned about the following:	Bathing practices. Frequency, the temperature of water, and the duration of bath	Postbathing care. The time of emollient application postbath, type and quantity of emollient, method of application of emollient, and type of clothing			Risk of food allergy		Use of diapers where applicable		Usage of steroids		Weather dependency of symptoms and signs
	The time of resolution of signs and symptoms										
In your clinical practice, how do you assess the immediate relief of signs and symptoms in babies with sensitive skin conditions including AD?	Child stops crying/is non-irritable	Child stops/reduces rubbing and scratching				Reduction in redness		Improvement in overall quality of life of child and/or family members			Others
As emollients help prevent the development of sensitive skin conditions, including AD, when would you ideally recommend the initiation of consistent emollient usage in babies?	From birth	From three months onward				From six months onward		Age-independent and dependent on the appearance of signs such as dryness and redness			Others
As per the latest available evidence, does the regular prophylactic application of colloidal oatmeal-based emollients, initiated in early infancy among high-risk infants, help prevent the occurrence and/or reduce the severity of AD?	Strongly agree	Agree				Neutral		Disagree			Strongly disagree

Table 5 shows the list of consensus statements that were excluded after round 2.

**Table 5:** A list of consensus statements that were excluded after round 2 (N = 7) Source: original table developed by the authors AD: atopic dermatitis

Number	Statement
1	The prevalence of sensitive skin tends to diminish with increasing age.
2	The regular usage of colloidal oatmeal-based emollients can reduce the severity of sensitive skin conditions, including AD, over time, by limiting disease progression in children.
3	Colloidal oatmeal-based moisturizers should be the cornerstone for the effective management of sensitive skin and conditions, including atopic dermatitis, and can be recommended for children with rosacea.
4	Colloidal oatmeal-based moisturizers should be the cornerstone for the effective management of sensitive skin and conditions, including atopic dermatitis, and can be recommended for children with damage from topical steroids.​
5	Colloidal oatmeal-based moisturizers should be the cornerstone for the effective management of sensitive skin and conditions, including atopic dermatitis, and can be recommended for children with seborrheic dermatitis and psoriasis.
6	Colloidal oatmeal-based moisturizers should be the cornerstone for the effective management of sensitive skin and conditions, including AD, and can be recommended for children with perioral dermatitis and photosensitive dermatoses.
7	Effective photoprotection in babies/children using an ideal sunscreen plays a pivotal role in safeguarding the skin against sensitivity, sunburns, and the exacerbation of underlying skin conditions.
